# Exercise Professionals with Advanced Clinical Training Should be Afforded Greater Responsibility in Pre-Participation Exercise Screening: A New Collaborative Model between Exercise Professionals and Physicians

**DOI:** 10.1007/s40279-018-0888-2

**Published:** 2018-03-09

**Authors:** Andrew J. Maiorana, Andrew D. Williams, Christopher D. Askew, Itamar Levinger, Jeff Coombes, Bill Vicenzino, Kade Davison, Neil A. Smart, Steve E. Selig

**Affiliations:** 1 Faculty of Health Sciences, School of Physiotherapy and Exercise Science, Curtin University, Kent St, Bentley, Perth, WA 6102 Australia; 2 Allied Health Department, Fiona Stanley Hospital, Perth, WA Australia; 3 School of Health Sciences, College of Health and Medicine, University of Tasmania, Launceston, TAS Australia; 4 Faculty of Science, Health, Education and Engineering, School of Health and Sport Sciences, University of Sunshine Coast, Sippy Downs, QLD Australia; 5 College of Sport and Exercise Science, Institute of Sport, Exercise and Active Living (ISEAL), Victoria University, Melbourne, VIC Australia; 6 Australian Institute for Musculoskeletal Science (AIMSS), Western Health, St Albans, VIC Australia; 7 School of Human Movement and Nutrition Sciences, Faculty of Health and Behavioural Sciences, University of Queensland, Brisbane, QLD Australia; 8 Faculty of Health and Behavioural Sciences, School of Health and Rehabilitation Sciences, University of Queensland, Brisbane, QLD Australia; 9 Division of Health Sciences, School of Health Sciences, University of South Australia, Adelaide, SA Australia; 10 School of Science and Technology, University of New England, Armidale, NSW Australia; 11 School of Exercise and Nutrition Sciences, Faculty of Health, Deakin University, Geelong, VIC Australia

## Abstract

Regular exercise improves health but can also induce adverse responses. Although such episodes are rare, many guidelines for pre-participation exercise screening have historically had a low threshold for recommending medical clearance prior to the commencement of exercise, placing the responsibility for decision making about exercise participation on physicians. The ‘clearance to exercise’ model still occurs widely in practice, but creates cost burdens and barriers to the uptake of exercise. Moreover, many physicians are not provided the training, nor time in a standard consultation, to be able to effectively perform this role. We present a model for pre-participation exercise screening and the initial assessment of clients wishing to commence an exercise programme. It is designed to guide professional practice for the referral, assessment and prescription of exercise for people across the health spectrum, from individuals who are apparently healthy, through to clients with pre-existing or occult chronic conditions. The model removes the request that physicians provide a ‘clearance’ for patients to engage in exercise programmes. Instead the role of physicians is identified as providing relevant clinical guidance to suitably qualified exercise professionals to allow them to use their knowledge, skills and expertise in exercise prescription to assess and manage any risks related to the prescription and delivery of appropriate exercise programmes. It is anticipated that removing unjustified barriers to exercise participation, such as mandated medical review, will improve the uptake of exercise by the unacceptably high proportion of the population who do not undertake sufficient physical activity for health benefit.

## Key Points

**Table:** 

Regular exercise is an important population health strategy, but a recommendation for medical clearance as part of pre-exercise participation screening can create an unjustified barrier to exercise.
Exercise professionals with advanced training in the prescription of exercise for pathological conditions are well-qualified to take greater responsibility for decisions about the suitability of clients to exercise.
The proposed model is likely to increase the uptake of best-practice exercise prescription and the associated health benefits.

## Introduction

Health professionals who prescribe exercise are faced with the so-called exercise paradox: while participation in regular physical activity is widely acknowledged to offer significant benefits to health and well-being [1–5], exercise can result in musculoskeletal injury [6] and induce symptoms or trigger adverse events for a wide range of chronic conditions [7, 8], including life-threatening cardiovascular events [9]. While, the risk of the latter is greatest for individuals with occult cardiovascular disease when undertaking unaccustomed activity [10], such occurrences are extremely rare [9, 11, 12] and the positive health outcomes of exercise vastly outweigh the risks of adverse signs, symptoms or events [13, 14].

To mitigate the potential risks of exercise, pre-exercise participation screening and risk assessment involving a review of the client’s medical history, and signs and symptoms indicative of underlying pathology, is advocated [15]. This usually involves a two-stage process: a pre-participation screening questionnaire, and, in individuals who are deemed at increased risk, referral to a physician (commonly a general practitioner [GP]) for clearance to exercise. However, there are several limitations to this approach. The threshold for recommending medical review has historically been low, resulting in an unjustified burden on the healthcare system [16] despite a lack of evidence that medical clearance mitigates the risk of cardiovascular events in asymptomatic individuals [17]. Additionally, the requirement to seek a medical appointment may create a barrier to the uptake of exercise [18], especially in individuals who are reticent to modify their lifestyle, and in socioeconomically disadvantaged groups in whom chronic disease is most prevalent and who have the most to benefit from regular exercise [19]. Despite the role expected of physicians, many medical school curricula do not address exercise screening and prescription in detail (in contrast to the training for exercise professionals [20]), meaning physicians are often not sufficiently familiar with exercise guidelines [21–23] and may feel ill-equipped to provide ‘clearance’ to exercise or the specific advice to offer about appropriate exercise parameters, such as type, intensity and duration [24, 25]. Importantly, the efficacy of medical clearance that is limited to an office assessment, constrained by a consultation of 15 min or less [26], in the absence of any assessment of the response to exercise is questionable.

We propose a model designed to reduce barriers to the uptake of exercise and improve the provision of best-practice exercise prescription for people across the health spectrum, consistent with current evidence that the benefits of exercise far outweigh the risks across a range of chronic health conditions [27]. In this model, suitably qualified exercise professionals take responsibility for the coordination of pre-participation exercise screening and use the findings to guide exercise prescription. This is done in conjunction with information and guidance from a physician when it is indicated, such as when a patient’s medical history highlights an existing chronic disease but further information is required, in the presence of new or progressive signs or symptoms, or when indications for undiagnosed disease exist. Critical to this approach is that exercise professionals manage clients who are consistent with their scope of practice and in whom they have the expertise to identify contraindications to exercise and subsequently inform individualised exercise prescriptions. We believe this approach will decrease the burden on health systems and more effectively utilise the skills of both exercise professionals and physicians to minimise the risk of adverse events during exercise, while maximising health benefits through increased exercise uptake and best-practice exercise prescription [28].

## Historical Models of Pre-Exercise Participation Screening

The American College of Sports Medicine (ACSM)/American Heart Association (AHA) Pre-participation Questionnaire (AAPQ) [28] and the Physical Activity Readiness Questionnaire (PAR-Q) developed by the Canadian Society for Exercise Physiology (CSEP) [29] are two highly utilised, but now superseded, pre-exercise screening models. The AAPQ classified individuals as high risk (established, or symptoms suggestive of, cardiovascular, pulmonary or metabolic disease), moderate risk (asymptomatic but with two or more cardiovascular risk factors) or low risk (asymptomatic with no more than one risk factor). The risk stratification process led to an algorithm that may recommend a medical examination before exercise was commenced. A recommendation may also have been made for a pre-participation exercise test, conducted either with or without physician supervision. Exercise prescription was then based on the assigned level of risk and the intensity at which the client wished to exercise.

The PAR-Q involved a series of ‘yes/no’ questions related to a history of, or symptoms suggestive of, cardiovascular or musculoskeletal conditions, and medication use. If the participant answered ‘yes’ to one or more questions they were advised to have a discussion with their medical practitioner before they became more physically active.

For many individuals, these processes created an unnecessary barrier to the uptake of exercise and increased burden on healthcare systems. A US study found that 95 and 68% of a nationally representative sample of US adults over 40 years of age [30] would be referred for medical review using the AAPQ and PAR-Q, respectively [16].

## Contemporary Models of Pre-Exercise Participation Screening

In view of the conservative approach of the traditional pre-exercise participation screening algorithm, in 2015 the ACSM published an updated statement on recommendations for pre-participation screening to reduce barriers to the uptake and maintenance of exercise and habitual physical activity [14] that considered several factors: that exercise is safe for most people; adverse responses to exercise are usually preceded by adverse signs or symptoms as early warnings; and the risks associated with exercise diminish as physical activity and fitness improve. Accordingly, it advised that pre-exercise screening should be guided by (1) current activity level; (2) the presence of signs, symptoms and/or known cardiovascular, metabolic or renal disease; and (3) the intensity of the intended exercise. In contrast to the historical recommendations, risk factor assessment is no longer included in the new pre-exercise participation screening process. The reader should refer to the ACSM recommendations for a detailed logic model for exercise participation based on this statement [14]. However, the recommendations maintain a requirement for ‘medical clearance’, and a recent evaluation of the new ACSM algorithm, using the same representative population sample as in the earlier study of the AAPQ [30], reported that 54% of respondents would still be referred for medical clearance before beginning any exercise [31].

The PAR-Q has also been revised in recent years to produce the PAR-Q+, through a comprehensive evaluation of the literature pertaining to the risks associated with exercise and physical activity [27]. The outcome of this review was the recommendation that in patients with existing cardiovascular disease, medical clearance is indicated only in individuals who are not medically stable, not currently physically active, and who have an aerobic power of < 5 Mets (< 17.5 ml kg^−1^ min^−1^), which is significantly different from the new ACSM algorithm and results in far fewer referrals for medical screening [32]. All other individuals are given the option of visiting a qualified exercise professional (with advanced university training) or their family physician.

In Australia, the Adult Pre-Exercise Screening System (APSS) was developed through a collaborative venture between the peak bodies for exercise science and exercise physiology, sports medicine, and personal training [33, 34]. The APSS is a three-stage screening tool, where Stage 1 identifies individuals with established disease or signs or symptoms of cardiovascular, metabolic/respiratory disease or musculoskeletal injury, and allocates a ‘high risk’ classification to all these groups. Stages 2 and 3 classify individuals as either moderate or low risk, based partly on the presence of common cardiovascular disease risk factors. These processes maintain elements of the outdated AAPQ and PAR-Q. ‘Low risk’ clears the individual to undertake exercise at any intensity without restriction, while those classified as moderate risk are cleared to perform low- and moderate-intensity exercise initially. Individuals who are classified as higher risk (from Stage 1), or those classified as moderate risk (from Stages 2 and 3) and wishing to undertake vigorous exercise, are advised to seek ‘guidance’ from a physician or appropriate allied health professional prior to commencing exercise. While the intent of this statement was to give greater responsibility to ‘appropriate allied health professionals’, these professionals were not clearly defined and it commonly leads to clients being sent to their physician for ‘clearance to exercise’, which has caused concerns within the medical profession [35].

## Methods

Exercise & Sports Science Australia (ESSA) is the peak body in Australia representing the various professionals working in the broad areas of exercise and sports sciences, and is responsible for accrediting university-educated exercise professionals without (exercise scientists) and with (clinical exercise physiologists) advanced university training in prescribing exercise in pathological conditions. In response to differences in recommendations for pre-exercise screening in the international literature, and a lack of clearly defined referral pathways discriminating between exercise professionals who do not have advanced university training, and those who do, the ESSA sought to develop a new model for exercise referrals to support the uptake of safe and effective exercise for people with and without chronic conditions.

An expert panel with extensive collective experience in developing and implementing national professional standards in clinical exercise and physiotherapy was formed to develop a framework to inform the referral processes and interprofessional management of those clients identified through pre-exercise screening as requiring further assessment and guidance prior to commencing exercise. Broad consultation occurred with key stakeholders, including representatives nominated by the Royal Australian College of General Practitioners, Cardiac Society of Australia and New Zealand, and Sports Medicine Australia, to define the nature of the problem. The expert panel then extensively reviewed existing systems internationally and related literature utilising the PubMed and Scopus databases up until 25 August 2017, and publications relating to screening for exercise prescription were searched. The papers retrieved were examined, and further relevant references obtained by reviewing cited articles and cross-referencing. Through an iterative process, draft models were established and feedback was sought from end users through a national presentation, from both the stakeholder representatives and independent expert reviewers. Ultimately, a model was developed that reflected good practice, maintained the intent of current pre-exercise screening recommendations [14, 27], and was subsequently endorsed by all panel members.

## A New Model to Strengthen Collaborations between Exercise Professionals and Physicians

The model is underpinned by the training and scope of practice of different exercise professions (Table 1). In Australia, clinical exercise physiologists and physiotherapists are accredited to provide clinical exercise services as an integral part of the national Medicare health system [36]. In this respect, these exercise professionals are considered independent health practitioners, who are competent to take responsibility for decisions about exercise prescription using both evidence and clinical reasoning. This removes the need for physicians to ‘clear’ their patients prior to being referred for exercise. Instead, bidirectional referral pathways between physicians and exercise professionals are encouraged [37]. Exercise professionals have a responsibility to refer patients to a physician if any symptoms/signs of concern are identified, and physicians are encouraged to provide information on active and inactive conditions, treatments/interventions, and signs and symptoms to appropriately trained exercise practitioners. The latter can then use the information to ‘guide’ the exercise programme. This model improves the transfer of information, strengthens clinical governance, and reduces patients’ barriers to exercise. Although the model outlined here fits with the independence afforded exercise professionals within the Australian context, it could easily be applied in other countries.

**Table 1:** Qualifications and scope of practice of different exercise professions, using the Australian context as an example

Profession	Minimum qualification	Advanced-level education and clinical practicum in exercise prescription for pathological conditions, including clinical placements	Target population for exercise assessments and prescription	Exercise delivery
Personal trainer	A recognised fitness qualification (i.e. Certificate or Diploma) or Bachelor’s degree not accredited by ESSA	No	Healthy populations	Healthy populations, clients with stable chronic conditions under the guidance of the client’s treating physician and/or a clinical exercise physiologist/physiotherapist
Exercise scientist	Bachelor’s degree in exercise science, with ESSA accreditation	No	Healthy populations	Healthy populations, clients with stable chronic conditions where a clinical exercise physiologist/physiotherapist has provided the exercise prescription
Clinical exercise physiologist,	Bachelor’s or Master’s degree in clinical exercise physiology	Yes	Healthy populations through to clients with chronic disease, including active cardiovascular, metabolic and renal disease	All clients free of absolute contraindications to exercise
Physiotherapist	Bachelor’s or Master’s degree in physiotherapy	Yes		All clients free of absolute contraindications to exercise

*ESSA* Exercise and Sports Science Australia

**Fig. 1:**
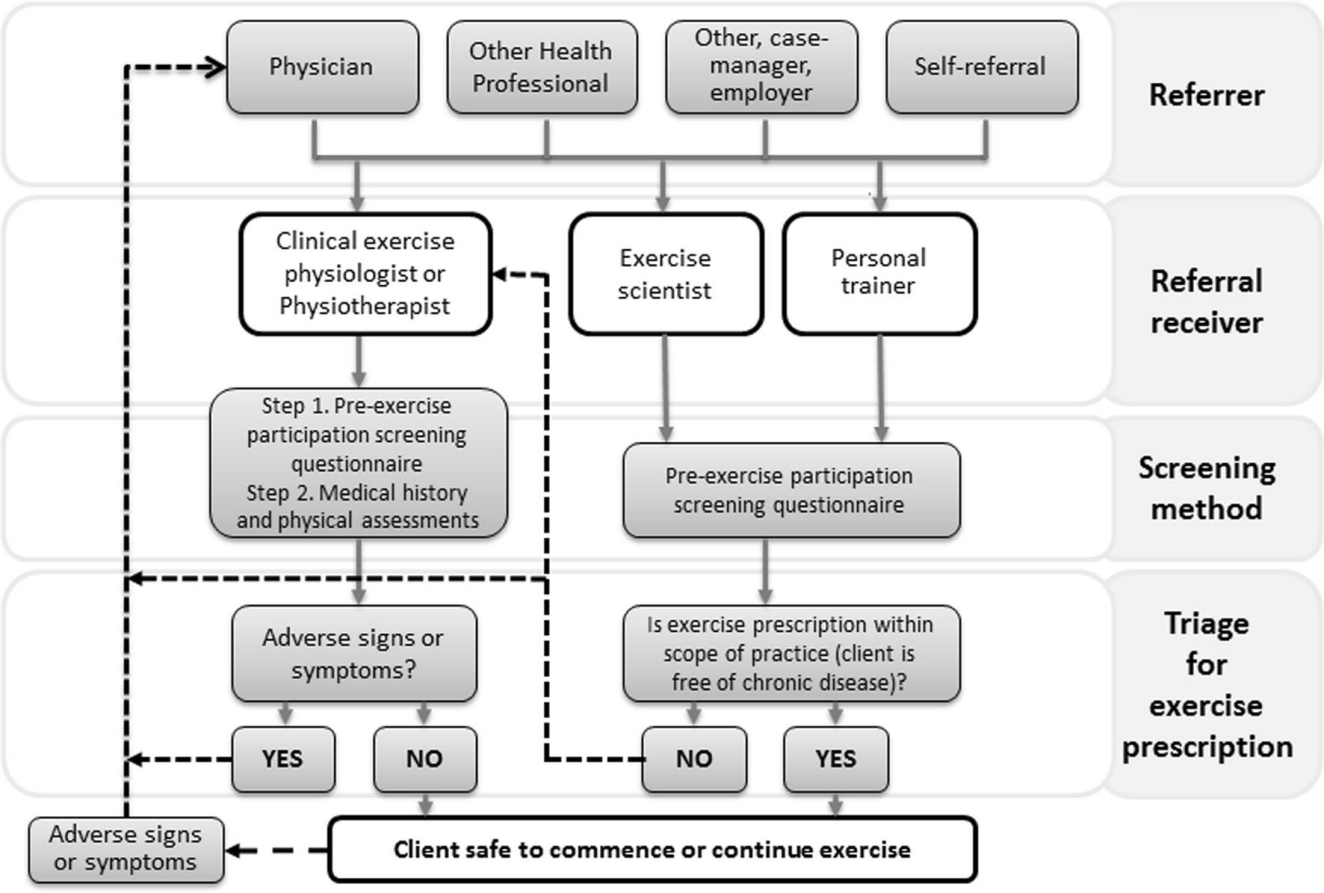
Referral and assessment pathways for guiding exercise prescription for individuals with and without chronic disease

The model considers three key components of the process for initiating exercise: referral, screening and triage (Fig. 1).

- Referral: exercise referrals may come from a variety of sources (including physicians, employers, rehabilitation case managers, other health professionals, and clients self-referring) and be received by any of the exercise professions. The referral should include as extensive a clinical history as is available, but the extent of this is likely to vary depending on the referral source, and additional information may need to be sought.
- Screening method(s): a pre-participation screening questionnaire is important to provide initial screening. However, when an existing chronic condition is known a priori, a clinical exercise physiologist/physiotherapist should apply advanced-level clinical reasoning, guided by a medical history and physical assessment to ascertain the necessary information for appropriate exercise prescription.
- Triage for exercise prescription: following screening, clients are triaged on the following basis:
  i. No history of chronic disease: exercise can be prescribed by any exercise professional.
  ii. Pre-existing chronic disease: The initial assessment and programme development should be conducted by an exercise professional with advanced training in clinical exercise prescription (clinical exercise physiologist or physiotherapist). In circumstances where a client with a chronic disease is demonstrated to be clinically stable under exercise conditions, the client may be transferred from a clinical exercise professional who has prescribed exercise to a non-clinical practitioner who supervises the client. However, oversight of ongoing safety of participation is managed by the clinical exercise professional.
  iii. The presence of adverse signs or symptoms: in the case of previously undiagnosed, or worsening, adverse signs or symptoms, the client should be referred to a GP and/or medical specialist for review and appropriate management prior to commencing/continuing exercise. These may be identified by a pre-participation screening questionnaire, during interview, or arise at the initial exercise assessment or during a subsequent exercise programme. When the physician considers the patient to be clinically stable, they should refer the client back to the exercise professional.
  iv. If a client’s clinical status is ambiguous: it is recommended that more detailed clinical information is sought from their physician before embarking on an exercise programme.

## Recommendations to Support Utilisation of the Model

The new model extends risk assessment beyond a brief review to prolonged observation under exercise conditions. The referral pathways share elements from contemporary models, such as those developed by the ACSM [14] and CSEP [27], and embrace the principles that guide them: that exercise is safe for most people; adverse responses to exercise are usually preceded by adverse signs or symptoms as early warnings (Table 2); and the risks associated with exercise diminish as habitual physical activity increases and fitness improves. Physicians are encouraged to play a proactive role in this process by providing information about patients under their care to clinical exercise physiologists or physiotherapists who can apply this in developing an individualised exercise programme. This information should include specific details about active and inactive conditions, treatments/interventions, and signs and symptoms experienced by the patient. Key recommendations are as follows:

**Table 2:** Common adverse signs and symptoms that are indications for the cessation of exercise

Sign or symptom
**Absolute indications for cessation of exercise**
Decrease in systolic blood pressure (from rest) ≥ 10 mmHg in the presence of symptoms
Development of significant ventricular or atrial arrhythmias
ST-segment depression (> 2 mm) or elevation (> 1 mm)
Shock or pacing therapies from implantable cardioverter defibrillator (ICD) or life vest
The onset of chest pain/discomfort, or other symptoms, suggestive of myocardial ischaemia
Dizziness, confusion, deteriorating balance or other significant neurological symptoms
Paleness or cyanosis
Vomiting, nausea or feeling generally unwell
Exhaustion or fatigue that is out of keeping with the person’s usual response to exercise at a given intensity
**Relative indications for cessation of exercise**
Decrease in systolic blood pressure from rest ≥ 10 mmHg in the absence of symptoms
Systolic blood pressure ≥ 250 mmHg and or diastolic blood pressure ≥ 115 mmHg
Increase in occurrence of ventricular ectopic beats with increasing intensity of exercise, including ventricular couplets, multifocal extrasystoles, bigeminy
Onset of supraventricular tachycardia or bradyarrhythmias
Onset of exercise-induced conduction defects
Atrial fibrillation that is inadequately rate-controlled with increasing exercise intensity
Chronotropic incompetence resulting in failure of heart rate to increase in response to exercise
Attainment of maximum predicted or prescribed heart rate or rating of perceived exertion
Onset or worsening of musculoskeletal pain
Limiting claudication
Wheezing or significant dyspnoea

This table is based on the indications for the termination of exercise testing as recommended by the American Heart Association [50]; however, situational clinical decision making is also important and may result in some modification of the application of the above criteria. Clinical decision making should include considerations of client factors; the nature of any medical referral; intensity, mode and volume of exercise; the qualifications, experience and competencies of the clinical exercise practitioner; the facility, including other staffing and equipment; and the capacity for providing life support

1. Physicians should provide guidance rather than ‘clear’ their patients for exercise: The concept of medical clearance, which implies that the physician has assessed their patient as ‘safe for exercise’, often based on an office assessment, is removed from the model. Instead, physicians should provide clinical guidance as indicated, given that appropriately trained exercise professionals have the requisite knowledge, skills and competencies to provide a safe and effective exercise service for the patient.
  The transfer of essential client information to guide safe and effective exercise prescription relies on clear communication between physicians and exercise professionals.
  Clinical exercise physiologists and physiotherapists undergo rigorous training, producing very high levels of minimum standards. In Australia, this credentialing occurs through independent accreditation schemes of national university coursework programmes. This benefits patients by them being able to access these services using a range of compensable schemes, including the government-funded Medicare programme.
2. Physicians retain their roles in recommending against exercise: if this is warranted based on the individual’s clinical status. Importantly, the proposed model is bidirectional, whereby if adverse signs or symptoms arise and are identified by the exercise professional during screening, or at some point during subsequent exercise sessions, the client should be referred back to their medical practitioner before exercise resumes.
  An important aspect of the model is that clients’ physicians are given ongoing information regarding their patient’s exercise participation so that they have the opportunity to provide guidance to the client and exercise professional as they see fit.
  Optimal medical management by physicians can alleviate symptoms and improve exercise safety, resulting in improved exercise tolerance and more effective exercise prescription [38–40]
3. Use clinical assessment and reasoning and/or questionnaires to guide exercise prescription: Non-clinical exercise professionals should use a contemporary pre-exercise screening questionnaire. For clients with known pathology, clinical exercise physiologists and physiotherapists should consult the patient’s medical history, review their exercise/physical activity history and conduct a physical assessment.
4. Patients should undergo an initial exercise assessment: Prior to designing and implementing an exercise intervention, exercise professionals should assess their clients’ exercise capacity.
  A baseline exercise assessment is an extension of the screening process, and, in some cases, signs or symptoms of undiagnosed or diagnosed pathology may become evident for the first time during an exercise assessment. In such cases, the test should be terminated and the client referred to their medical practitioner for further clinical evaluation and treatment.
  It is important to distinguish between physician-supervised exercise stress tests and other forms of exercise assessments employed by exercise professionals. To clarify, a physician-supervised exercise stress test would be indicated for symptomatic individuals or those with suspected pathology. It aims to provoke adverse signs or symptoms in order to contribute to the diagnosis of cardiovascular disease or other conditions [41]. In contrast, tests administered by exercise professionals are generally designed to determine a client’s fitness so that an exercise programme can be appropriately prescribed within a range of exercise intensities that are below any threshold for adverse signs or symptoms.
5. Clinical reasoning based on all observations should then be used to guide exercise prescription:
  Clinical status and current exercise levels are used to guide commencing and maximal exercise training intensity (Table 3).

**Table 3:** Recommended exercise training intensity range and exercise testing protocol according to the clinical status of clients

Clinical status	Currently exercising?	Initial exercise intensity	Maximum exercise intensity
No existing or suspected chronic disease	Yes	Usual exercise intensity	Progress up to vigorous intensities as exercise tolerance allows
	No	Light—moderate	Progress up to vigorous intensities as exercise tolerance allows
Existing/suspected chronic disease	Yes	Moderate intensity	Moderate some clients may progress to vigorous intensities after careful assessment
Undiagnosed signs or symptoms suggestive of unstable chronic disease	No	Light–moderate	Moderate some clients may progress to vigorous intensities after careful assessment
	Yes or no	Clients should avoid structured exercise until diagnosed by a medical practitioner or cleared of disease	NA

Classifications of exercise intensity are indicated as those described by Norton and colleagues [51]
  Exercise should commence conservatively (no greater than moderate intensity) for previously sedentary clients who are starting an exercise programme. This is likely to reduce the potential for injuries as well as adverse cardiovascular responses [10]. Exercise can routinely proceed to moderate intensities for clients with stable chronic disease as the incidence of adverse events is very low [42–44].
  For clients with cardiovascular disease who wish to undertake vigorous exercise, clinical reasoning should be carefully applied by an experienced clinical exercise physiologist or physiotherapist, and informed by advanced risk assessment algorithms [45]. Progression to vigorous exercise should be determined on a case-by-case basis, guided by the client’s medical history and current exercise tolerance. In some patients with established cardiovascular disease, a physician-supervised exercise stress test (± radionuclide scanning) may help inform a decision about whether vigorous-intensity exercise is appropriate or not. Some clients with stable cardiovascular disease, normal left ventricular ejection fraction, no significant residual coronary lesions and good exercise tolerance should be able to progress quite safely to vigorous exercise [46, 47]. There have been recent encouraging findings for the efficacy of high-intensity interval training (HIIT) in people with cardiometabolic disease [42, 44, 48], although preliminary comparisons showing that the rate of adverse responses are increased during HIIT, compared with moderate-intensity exercise, emphasises the need for caution [43, 49].
  Exercise prescription is individualised and flexible, adapting with the patient’s fitness. In this way, the benefits of exercise can be achieved with progressive adaptation and least risk.
6. Exercise professionals should provide regular feedback to the referring physician. This should include a description of the exercise prescription in practical terms so that the physician can provide a consistent message to the patient/client about desired exercise intensity and restrictions.

## Conclusions

We present a new model of collaboration between exercise professionals and physicians that reflects contemporary evidence related to the risks and benefits of exercise, and utilizes the collective expertise of these professions to improve the uptake and maintenance of safe and effective exercise. The model encompasses the requirements of clients with and without chronic disease and encourages the application of exercise screening, based on contemporary guidelines, by appropriately qualified exercise professionals. For patients with a chronic disease, exercise screening and prescription should be performed by an exercise professional with education and training in pathological states and their significance to the exercise response (clinical exercise physiologists, physiotherapists). This necessitates bidirectional referral and communication pathways with the client’s physician. The role of physicians remains critical, by providing details of medical history to help inform appropriate exercise prescription, diagnosing and treating new signs and symptoms, and offering guidance to exercise professionals based on a determination of the clinical requirements of their patients.
